# Neonatal Cholestasis Progressing to a Multisystem Syndrome With Liver Cirrhosis in Two Siblings With FARSA Deficiency: An Evolving Hepatological Phenotype

**DOI:** 10.1002/jmd2.70013

**Published:** 2025-04-04

**Authors:** Y. Aelvoet, P. Verloo, A. Vanlander, S. Vande Velde, S. Van Biervliet, P. De Bruyne, L. Hoste, A. Dheedene, L. Pottie, A. Hoorens, M. Mendes, R. De Bruyne

**Affiliations:** ^1^ Department of Pediatrics Princess Elisabeth Children's Hospital Ghent Belgium; ^2^ Faculty of Medicine and Health Science Ghent University Ghent Belgium; ^3^ Department of Pediatric Neurology and Metabolic Diseases Princess Elisabeth Children's Hospital, Ghent University Hospital Ghent Belgium; ^4^ Department of Pediatric Gastroenterology, Hepatology and Nutrition Princess Elisabeth Children's Hospital Ghent Belgium; ^5^ Specialized Pediatric Ward Princess Elisabeth Children's Hospital, Ghent University Hospital Ghent Belgium; ^6^ Center for Medical Genetics Ghent University Hospital Ghent Belgium; ^7^ Department of Pathology Ghent University Hospital Ghent Belgium; ^8^ Department Laboratory Medicine, Laboratory Genetic Metabolic Diseases Amsterdam UMC, University of Amsterdam Amsterdam the Netherlands; ^9^ Amsterdam Gastroenterology Endocrinology Metabolism Amsterdam UMC, University of Amsterdam Amsterdam the Netherlands

**Keywords:** FARSA deficiency, idiopathic liver disease, neonatal cholestasis, neonatal liver failure, phenylalanyl‐tRNA, Rajab interstitial lung disease, tRNA synthetase

## Abstract

Biallelic variants in *FARSA* or *FARSB* are associated with reduced cytoplasmic phenylalanyl‐tRNA synthetase (FARS1) activity and underlie a multisystem syndrome characterized by growth limitation, developmental delay, brain calcifications, interstitial lung disease (ILD), and liver involvement. ILD is an early characteristic feature marked by bilateral ground‐glass opacification, subpleural cysts, and cholesterol pneumonitis and seems to be the leading cause of disease burden and death. A 7‐year‐old Iraqi girl was referred with idiopathic liver disease. Her previous medical history revealed neonatal jaundice, failure to thrive (FTT), mild motor development delay, and variceal bleeding at the age of 6 years in Iraq. She was diagnosed with liver cirrhosis, severe splenomegaly, profound thrombocytopenia, and hypoalbuminemia. Her younger brother presented to our hospital at the age of 2 months with neonatal cholestasis progressing to hepatic failure with impaired synthetic function. He suffered from coagulopathy, intractable hypoalbuminemia, FTT with axial hypotonia, multiple infectious episodes, and a prothrombotic state. Whole exome sequencing revealed compound heterozygous missense variants p.(Pro226Leu) and p.(Arg475Trp) in *FARSA* (OMIM: 602918) in both siblings. Even in the absence of overt clinical symptoms, chest computer tomography following diagnosis showed ILD in both siblings. Decreased FARS1 activity was measured in fibroblasts of both patients. We are the first to report on two siblings with neonatal jaundice evolving to severe liver disease as a cardinal symptom of cytosolic FARS deficiency. We emphasize the importance of performing a pulmonary workup in the diagnostic process of liver failure of unknown origin for detection of ILD as a clue to diagnosis.

1

**Summary**

- First report of a heterogeneous hepatological phenotype with neonatal jaundice and neonatal liver failure in two siblings with *FARSA* deficiency evolving to liver cirrhosis, as the main symptom of multisystem disease in association with growth restriction, motor delay and asymptomatic ILD.

## 2 Introduction

Biallelic mutations in the cytoplasmatic phenylalanyl‐tRNA synthetase (FARS1) subunit genes *FARSA* and *FARSB* can cause a severe and early‐onset multisystem neurodevelopmental disorder with developmental delay, significant growth limitation, brain calcifications, interstitial lung disease (ILD), and liver dysfunction as key clinical symptoms [1, 2, 3, 4, 5, 6, 7, 8, 9, 10, 11]. FARS1 is a tetrameric protein containing two catalytic alpha (FARSA) and two regulatory beta (FARSB) subunits. The enzyme catalyzes an essential step of protein biosynthesis by covalently charging tRNA^Phe^ with phenylalanine in the cytoplasm [1, 5, 12]. In patients' fibroblasts or EBV transformed lymphoblastoid cell lines (LCLs), aminoacylation activity is impaired but not absent. The latter is suspected to be incompatible with life [5, 12].

Patients with biallelic pathogenic variants in other cytoplasmatic aminoacyl‐tRNA synthetase (ARS1) genes share clinical features. Patients with variants in *CARS1*, *IARS1*, *LARS1*, *MARS1, VARS1, YARS1*, *FARSA*, and *FARSB* have been documented to suffer from liver disease and failure to thrive (FTT) [9, 10, 11, 12, 13, 14, 15, 16]. This phenotypic overlap suggests that mutations in different ARS1 genes have a significant common impact on protein synthesis via the ligation of amino acids to their cognate tRNA [1, 13, 14, 15, 16].

To the best of our knowledge, only 11 individuals have previously been reported with a *FARSA*‐related syndrome [3, 5, 9, 10]. However, pathogenic variants in both FARS1 subunits coding genes *FARSA* and *FARSB* are associated with a similar clinical syndrome [5]. Both subunits form a hetero‐tetramer, and mutations in both *FARSA* or *FARSB* cause decreased FARS1 activity, implying that clinical similarity is expected [8, 12]. Until now, 18 patients are described in the literature with *FARSB*‐related disease [1, 5, 6, 8, 11]. Common features observed in affected FARS1 patients are ILD, neurodevelopmental delay, FTT, abnormal liver biochemical tests, and hypoalbuminemia. Additional respiratory characteristics (cystic lung disease and digital clubbing), abnormal neurological findings (brain cysts and calcifications), liver involvement (hepatosplenomegaly, liver steatosis, fibrosis or cirrhosis) and connective tissue, vascular and muscular abnormalities have been described previously [5].

ILD is an early hallmark feature of FARS1 deficiency associated with recurrent respiratory infections, chronic cough, or dyspnea and is among the leading causes of disease burden and mortality [5, 9, 10]. High resolution chest computer tomography (CT) typically shows bilateral ground‐glass opacification, septal thickening, and subpleural cysts. Cholesterol pneumonitis and infiltration of inflammatory cells are characteristic histological findings [5].

## 3 Case Report

Here, we describe the cases of a meanwhile 14‐year‐old girl (with a follow‐up of six and a half years) and her 3‐year‐old brother. The family consists of five, with two healthy, non‐consanguineous Iraqi parents and one healthy sister. No particular familial diseases were noted (Figure S1).

### 3.1 Clinical Presentation

#### 3.1.1 Patient 1

The oldest sibling, a girl (P1), presented to our center at the age of 7 years with idiopathic liver cirrhosis and significant portal hypertension. From her previous history, neonatal jaundice, FTT, and rickets were noted at the age of 6 months. Due to follow‐up in an Iraqi hospital, we did not have access to detailed medical information, but she remained hospitalized after birth for several weeks because of neonatal jaundice. She suffered from an esophageal variceal bleed at the age of 6 years requiring band ligation in Iraq. Gross motor milestones were moderately delayed with independent walking at the age of two. There were no overt respiratory symptoms.

Clinical examination on the first encounter at our clinic revealed a frail girl (growing on the 12th centile for weight and height, Figure S2.1) with significant muscle wasting, severe clubbing, a protuberant abdomen, and manifest splenomegaly. There was severe thrombocytopenia with leukopenia and normal red blood cell counts (Figure S3). Serum transaminases, gamma‐glutamyltransferase, bilirubin, albumin, and coagulation were all within normal values.

**FIGURE 1:**
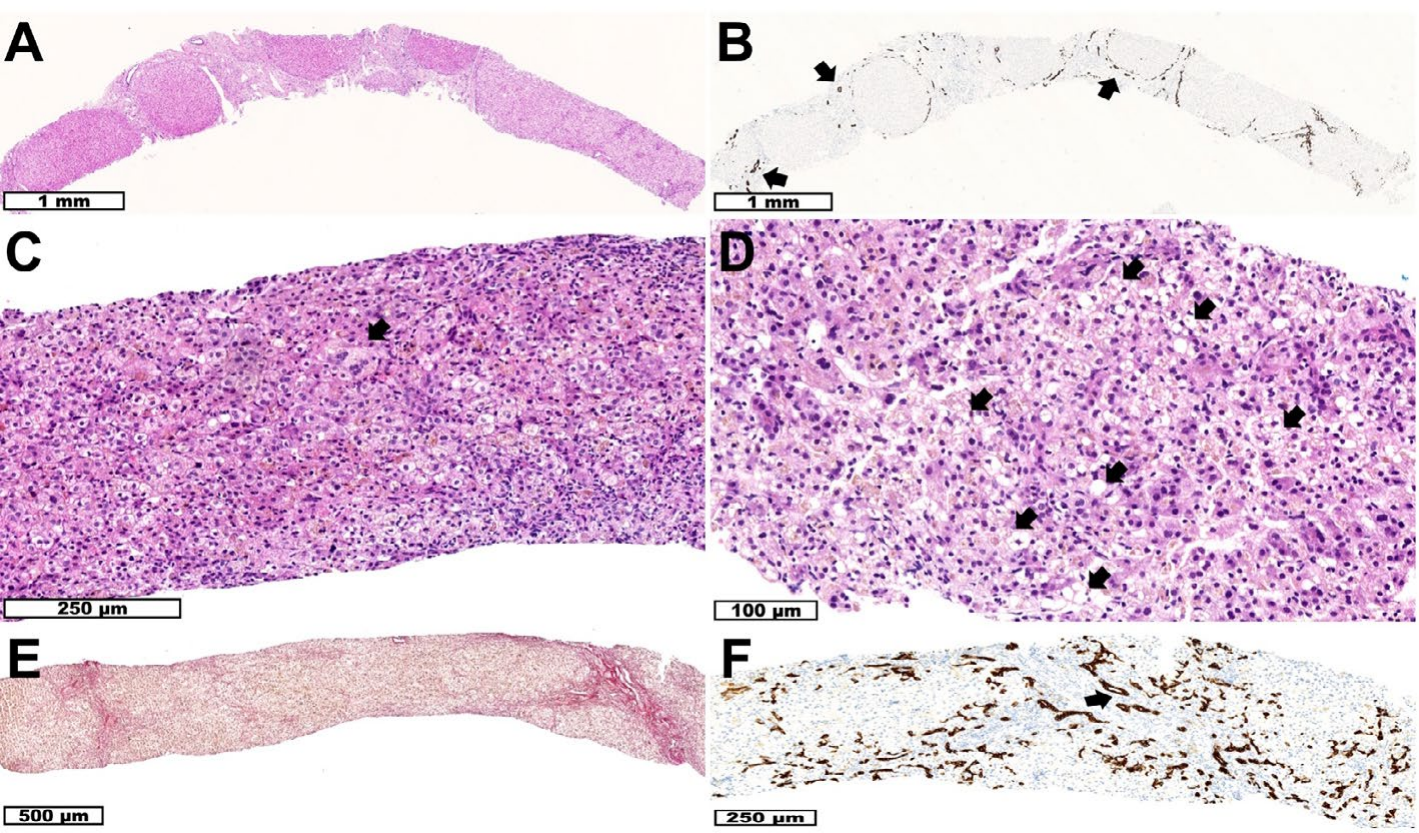
Liver histology. P1: (A) Liver biopsy shows cirrhosis. (HE staining) (B) Keratin 7 immunohistochemistry shows preserved interlobular bile ducts (arrows) and mild ductular reaction. P2: (C) Liver biopsy shows severe bilirubinostasis with few multinucleated giant cell hepatocytes (arrow) without significant inflammation. (HE staining) (D) Higher magnification in addition shows microvesicular steatosis (arrows). (HE staining) (E) Sirius red staining shows beginning septal fibrosis. (F) Keratin 7 immunohistochemistry shows preserved interlobular bile ducts (arrows) and moderate ductular reaction.

Cardiac ultrasound and sweat test for excluding cystic fibrosis were normal. On abdominal imaging, she had a cirrhotic aspect of the liver with a large caudate and left hepatic lobe and significant splenomegaly with a T2 hypo‐intense nodule cranially in the spleen and extensive collateralisation due to portal hypertension. Because of severe thrombocytopenia (< 4000/μl), a bone marrow biopsy was performed, revealing a normal cell‐rich bone marrow. Liver biopsy showed cirrhosis with a discrete lymphocytic inflammatory infiltrate in the fibrous septa, preserved interlobular bile ducts, and mild ductular reaction without ductular metaplasia of periportal hepatocytes (Figure 1A,B). Histologically, there were no etiological clues. Further comprehensive workup for underlying chronic liver disease, including microarray and panel‐based (in‐house intellectual disability (ID) and epilepsy and hepatology panel) exome sequencing, did neither lead to any diagnostic clues.

#### 3.1.2 Patient 2

Her brother (P2) was referred at the age of 2 months due to neonatal cholestasis and progressive liver dysfunction. He was born term after a pregnancy complicated by diabetes gravidarum. Birth weight was 3.390 kg (40th centile, −0.3 SD) and length 51 cm (52th centile, 0.0 SD). The neonatal course was complicated with substantial jaundice and axial hypotonia. Upon admission, the patient was found to have faltering growth (weight 4.140 kg; 1st centile, −2.4 SD and length 55 cm; 7th centile, −1.5 SD, Figure S2.2). On initial abdominal palpation, there was no apparent organomegaly, but a left‐sided inguinal hernia was present. Hypoalbuminemia, elevated transaminase levels, and increased direct bilirubin were seen upon admission, as well as anemia and thrombocytopenia. Laboratory findings during initial hospitalizations are shown in Figure S4. The boy developed ascites and refractory hypoalbuminemia, contrasting with a modest severity of liver failure, initially requiring multiple intravenous albumin transfusions in association with diuretic treatment.

**FIGURE 2:**
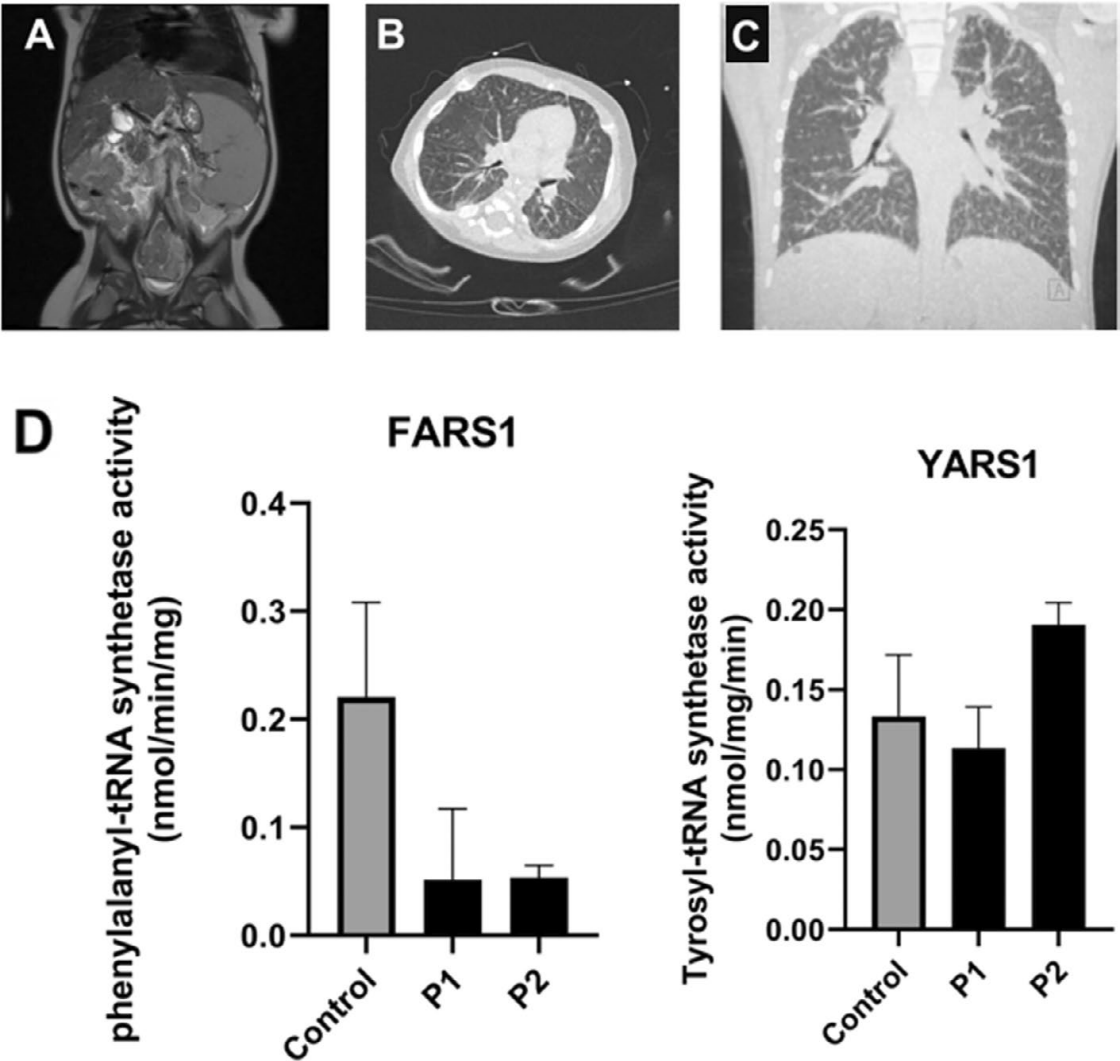
Imaging. (A) Abdominal MRI (T2) of P2 demonstrating heterogeneous liver parenchyma and severe splenomegaly. (B) HRCT of the lungs of P2 showing global ground‐glass opacification. (C) HRCT of P1 showing diffuse interstitial lung disease with thickened interlobar septa, peripheral subpleural reticulations, micronodular distention of lung fissures, and the presence of several lung (micro)nodules. (D) FARS1 and YARS1 activity in fibroblasts of a healthy control and both siblings, showing decreased FARS1 activity in the FARSA patients.

Abdominal imaging (ultrasound and magnetic resonance imaging (MRI); Figure 2A) demonstrated hepatic cirrhosis with highly heterogeneous liver parenchyma containing many small nodules (without diffusion restriction), severe splenomegaly, repermeabilization of the umbilical vein, and ascites. An extensive workup for neonatal cholestasis including viral serology, TORCH (toxoplasmosis, rubella, cytomegalovirus, herpes simplex, and HIV) screen, sweat test, and extensive metabolic assays (including but not limited to GALT (galactose‐1‐phosphate uridyltransferase) activity, plasma amino acids, urinary organic acids, plasma acylcarnitine profiling, and urinary polyol analysis) did not explain the clinical picture. Liver biopsy showed severe bilirubinostasis, few multinucleated giant cell hepatocytes, and incipient acinar transformation of the liver parenchyma, microvesicular steatosis, and beginning septal fibrosis (Figure 1C–F).

The patient suffered from multiple life‐threatening infectious episodes, including recurrent bacteremia ( *C. parapsilosis* and *E. coli* ) and cytomegalovirus (CMV) enterocolitis with severe diaper dermatitis and impaired wound healing. These episodes were treated with caspofungin, piperacillin‐tazobactam, and valganciclovir, in addition to supportive measures such as rehydration, correction of electrolyte disturbances, and administration of immunoglobulins.

A prothrombotic state with the formation of multiple thrombi in deep vessels led to serious venous access problems. At 4 months of age, the boy developed persistent diarrhea, vomiting, and skin rash due to cow's milk allergy (confirmed by elimination and provocation test). Elimination of cow's milk protein did not affect the refractory hypoalbuminemia. There was renal tubulopathy with intercurrent proteinuria, elevated urinary alpha‐1‐microglobulin levels requiring supplementation of sodium bicarbonate as well as persistent hypoglycemia necessitating enteral maltose supplementation. Growth hormone and glucagon tests were normal. Cardiac ultrasound showed an atrial septal defect (ASD) type II. Eye examination and hearing tests were normal. Chest CT (Figure 2B) demonstrated global ground‐glass opacification without cystic lung lesions, emphysema, or areas of confluence which may suggest early signs of ILD. Overnight oximetry was normal. A small subependymal cyst and some minimal white matter lesions were noted on a brain MRI.

As both siblings had a similar clinical presentation, quad whole exome analysis was performed. In both siblings, compound heterozygous missense variants c.677C>T p.(Pro226Leu) and c.1423C>T p.(Arg475Trp) in *FARSA* (NM_004461.2) were found, with confirmation that each parent carried one of these variants. Both identified variants are rare, with AF (allele frequency) of 0.0013% (21 exome allele count) and 0.0035% (54 exome allele count) in the gnomAD v.4.1.0 database, respectively. Neither variant has been previously reported in ClinVar. The p.(Arg475Trp) variant has been observed in one patient with FARSA with respiratory infections and liver dysfunction, where it co‐occurred with a different variant p.(Pro347Leu) in a compound heterozygous state (PMID: 35918773) [7].

To confirm the pathogenic nature of the found variants, aminoacylation activities for FARS1 and YARS1 (cytosolic tyrosyl‐tRNA synthetase) were measured in fibroblasts of both siblings, as described by Mendes et al. [12] In P1 and P2, residual FARS1 activity was 27% and 28% of controls, respectively, while YARS1 activity was comparable to controls (Figure 2D).

Following the diagnosis of FARS1 deficiency P1 underwent chest CT (Figure 2C) demonstrating diffuse ILD with thickened interlobar septa, peripheral subpleural reticulations, micronodular distention of lung fissures, and the presence of several lung (micro)nodules. Pulmonary function testing shows restrictive lung disease (decreased force vital capacity (FVC 53%) with a normal Tiffeneau‐index (FEV1/FVC 88%)). Subependymal cysts and intracranial calcifications were not found on brain MRI.

Due to the severe clinical condition and the absence of other therapeutic options, a trial with phenylalanine substitution was started (70–80 mg/kg/d) in both siblings. Surveillance following the initiation of Phe substitution reveals a transient and slight increase in phenylalanine levels in the plasma in both siblings (e.g., 61–112 μmol/L in P1, 51–85 μmol/L in P2). Currently, a 3‐year follow‐up of phenylalanine supplementation shows P1 in a stable condition. However, she has a severely decreased exercise tolerance based on restrictive pulmonary disease, and severe thrombopenia remains present (Figure S3). Her younger brother has a favorable clinical evolution with catch‐up growth and a reduction in the need for albumin supplementation (Figure S4). Albumin was administered more than once a week throughout the first 3 months after admission, followed by two to four albumin transfusions per month for 3 months and three transfusions in the following months. Final albumin suppletion was given at the age of 12 months, 6 months after phenylalanine supplementation was initiated. Screening gastroduodenoscopy in the context of portal hypertension revealed Grade 1 esophageal varices at the gastroesophageal junction.

## 4 Discussion

With the clinical cases described above, we add two patients to the small international cohort of FARS1 patients. In doing so, we extended the clinical presentation by adding profound neonatal cholestasis and neonatal liver failure, not previously reported. To date, FARS1 patients described in the literature were diagnosed due to FTT and growth retardation, truncal hypotonia, delay in developmental milestones, and respiratory insufficiency [1, 2, 3, 5, 8]. In the majority of patients, pulmonary involvement is characterized by tachypnea, chronic cough, and recurrent respiratory tract infections manifesting early in life [1, 2]. Further examination performed revealed liver abnormalities, consisting of increased transaminases, mild/moderate steatosis, fibrosis or cirrhosis, and/or hypoalbuminemia upon admission or during follow‐up [1, 2, 3, 5, 8].

An overview of the clinical features in our and previously reported patients with *FARSA* or *FARSB* mutations described in the literature is summarized in Table 1 (Detailed information in Table S1).

**TABLE 1:** An overview of the clinical features in our and previously reported patients with *FARSA* or *FARSB* mutations.

	P1 (girl)	P2 (boy)	FARSA (incl P1–2) (*n* = 13)	FARSB (*n* = 17)	FARS1 (%)
Respiratory					
Interstitial lung disease	X	X	13/13	16/17	97%
Cystic lung disease			6/13	6/17	40%
Cholesterol pneumonitis (lung biopsy)			6/9	7/7	81%
Pulmonary alveolar proteinosis			2/13	1/17	10%
Intra‐alveolar hemorrhage			2/13	0/17	7%
Chronic cough			4/13	4/17	27%
Recurrent spontaneous pneumothorax			0/13	3/17	10%
Digital clubbing	X	X	7/13	4/17	37%
Nervous system					
Delayed motor development	X		8/13	11/17	63%
(Neonatal) hypotonia		X	8/13	6/17	47%
Speech delay			4/13	5/17	30%
Intellectual disability, learning difficulties			4/13	8/17	40%
Headache, migraine			1/13	8/17	30%
Seizures			0/13	4/17	13%
Microcephaly			4/13	9/17	43%
Extrapyramidal symptoms			0/13	3/17	10%
Brain cysts (MRI)		X	3/13	0/15	11%
Brain calcifications (MRI)			3/13	12/15	54%
White matter and gliotic lesions (MRI)		X	5/13	3/15	29%
Brain atrophy (MRI)			2/13	1/15	11%
Brain aneurysm or elongated arteries (MRI)			2/13	2/15	14%
Brain hemorrhage/stroke			2/13	2/17	13%
Hydrocephalus/ventriculomegaly			1/13	3/17	13%
Hypopituitarism			1/13	0/17	3%
Musculature and growth					
Decreased muscle mass	X		4/13	10/17	47%
Abnormal muscle histology			1/1	3/5	67%
Failure to thrive, poor weight gain	X	X	13/13	15/17	93%
Growth hormone resistance/deficiency			2/13	0/17	7%
Short stature	X	X	10/13	7/17	57%
Hepatobiliary					
Hepatomegaly, splenomegaly	X	X	10/13	3/17	43%
Elevated transaminases (AST, ALT)		X	8/13	3/17	37%
Elevated cholestasis parameters (gGT, bilirubin)		X	5/13	1/17	20%
Liver steatosis, fibrosis, cirrhosis	X	X	10/12	4/10	64%
Neonatal jaundice	X	X	3/13	0/17	10%
Gastrointestinal					
Feeding intolerance/difficulties		X	4/13	2/17	20%
Recurrent vomiting and/or diarrhea		X	6/13	4/17	33%
Inguinal hernia		X	2/13	2/17	13%
Intestinal malrotation			0/13	2/17	7%
Gastroesophageal reflux			0/13	2/17	7%
Gastroesophageal varices/hemorrhage	X	X	2/13	1/17	10%
Cardiovascular					
Structural heart or vessel defects		X	3/13	1/17	13%
Arterial hypertension		X	1/13	2/17	10%
Urinary					
Vesicoureteral reflux			1/13	1/17	7%
Proteinuria		X	2/13	3/17	17%
Renal artery stenosis			0/13	1/17	3%
Hyperphosphaturia			1/13	0/17	3%
Glomerulosclerosis (renal biopsy)			0/13	1/17	3%
Tubulopathy		X	3/13	0/17	10%
Nephrolithiasis			2/13	0/17	4%
Renal hyperechogenicity (ultrasound)			1/13	1/17	7%
Hematologic					
Anemia (microcytic)		X	4/13	8/17	40%
Thrombocytopenia	X	X	3/13	1/17	13%
Leukopenia, neutropenia	X		2/13	1/17	10%
History of DVT		X	1/13	1/17	7%
Metabolic					
Hypoalbuminemia	X	X	12/13	5/17	57%
Hypocalcemia			1/13	1/17	7%
Hypertriglyceridemia			1/13	1/17	7%
Hypoglycemia		X	1/13	1/17	7%
Neonatal rachitis	X		1/13	1/17	7%
Dysmorphism and skeletal					
Facial dysmorphism (Marfan‐like)			7/13	6/15	46%
Chest deformity (pectus excavatum/carinatum)			5/13	3/15	29%
Scoliosis			1/13	2/15	11%
Osteopenia		X	3/13	7/15	36%
Other systems					
Poor wound healing		X	2/13	1/17	10%
Abnormal subcutaneous fat tissue distribution			2/13	3/17	17%
Abnormal eye movements, nystagmus			1/13	1/17	7%
Sensorineural hearing impairment			1/13	1/17	7%
(Congenital) hypothyroidism			3/13	1/17	13%

In contrast to previously reported cases, we report two siblings with severe liver disease as the main symptom before overt clinical respiratory symptoms were present. P1 was diagnosed with idiopathic liver cirrhosis and significant portal hypertension after suffering from a variceal bleed requiring band ligation. P2 was referred because of neonatal cholestasis with progressive liver failure with coagulopathy, and liver cirrhosis on histology. Pulmonary examination following diagnosis revealed mild diffuse ILD in both siblings.

Respiratory failure is the main cause of morbidity and mortality in FARS1 deficient children [1, 5]. Of the 30 patients described in the literature, 12 died, 10 of whom were suffering from severe respiratory failure in childhood [1, 5, 7, 8, 9, 10]. One patient died due to pulmonary hypertension leading to a cardiac arrest at 8 years old, another due to an intracranial hemorrhage caused by a ruptured aneurysm at the age of 10 years [6]. Other less common life‐threatening complications were variceal bleeding, severe infectious episodes, catheter‐related venous thrombosis, and seizures [5].

The biochemical problem in aminoacyl‐tRNA synthetase deficiencies is at least partly due to reduced incorporation of the affected amino acid in proteins. Supplementation with the amino acid could give a rescue of incorporation. A protein‐rich diet and phenylalanine supplement might be beneficial in the treatment of FARS1‐related diseases; however, evidence is still very low [8]. Schuch et al. [5] described progressive lung disease despite a protein‐rich diet and phenylalanine supplement. One patient had a stable but low lung function at the last report, and clinical evaluation for transplantation was performed; another patient died despite diet and supplementation [5]. Our siblings' clinical condition has remained stable since phenylalanine supplementation was initiated. The girl, however, shows a non‐recuperating decreased exercise tolerance and refractory thrombopenia. Her younger brother shows mild gross motor delay but is generally in good health.

FARSA patients were recently found to have a chronic inflammatory profile with elevated CRP, leukocytosis, and high serum cytokine levels during episodes without infections. Evidence of autoimmunity due to positive rheumatoid factor, anti‐nuclear antibodies (ANA), and anti‐neutrophil cytoplasmic antibodies (ANCA) was also described [2]. Charbit‐Henrion et al. [2] reports the use of a JAK1/2 inhibitor because of positive interferon scores, with induced transient remission of inflammatory symptoms in one patient and possible protection by a preemptive treatment regimen in another patient. Initiation in two patients with late‐stage pulmonary fibrosis, however, shows a lack of efficiency likely due to advanced lung disease [2]. It could be hypothesized that intracellular stress mediated by defective cytosolic phenylalanyl‐tRNA synthetase pathways triggers interferon release and underlies some of the phenotypic features that are associated with multisystem inflammation. Such a pathophysiological mechanism might be similar to known interferonopathies, many of which share clinical features with FARS1 deficient patients such as brain calcifications, vasculopathy, ILD, and cytopenia. Although hepatic involvement is reported in this heterogeneous group of diseases, severe liver dysfunction, including refractory hypoalbuminemia, is not a hallmark symptom, suggesting that alternative pathways are involved. Finally, further research would be needed as this may give rise to new therapeutic possibilities, including further rationale for the use of interferon blocking treatments.

## 5 Conclusion

Biallelic pathogenic variants in *FARSA* and *FARSB* cause a similar multisystem disorder with growth failure, developmental delay, ILD, and hepatic abnormalities due to impaired aminoacylation activity [5, 8]. Respiratory failure is the leading cause of morbidity and mortality among affected infants and children.

We report the first case of neonatal jaundice and neonatal liver failure with coagulopathy and refractory hypoalbuminemia with progression to liver cirrhosis, without respiratory involvement, in two siblings with FARSA deficiency. We conclude that neonatal jaundice and severe liver disease can be a cardinal symptom of FARS1‐related disorders before ILD becomes apparent. In neonates and children with cryptogenic liver disease, a pulmonary workup hence needs to be considered to rule out ILD. The current treatment is limited to phenylalanine supplementation, though its effectiveness is uncertain [5, 8]. The presence of a systemic inflammatory profile needs further exploration, as it could explain life‐threatening phenotypic symptoms such as ILD and may therefore have significant implications for clinical management.

## Author Contributions

Y.A. and P.V. collected clinical data and drafted the initial manuscript. P.V., R.D.B., and L.H. critically reviewed and revised the manuscript. Y.A., P.V., R.D.B., A.V., S.V.V., S.V.B., and P.D.B. were directly involved in the patient's care and contributed through critical review and revision of the manuscript. A.D., L.P., A.H., and M.M. were responsible for collecting anatomopathological and biochemical data. All the authors approved the final manuscript as submitted.

## Consent

All procedures followed were in accordance with the ethical standards of the responsible committee on human experimentation (institutional and national) and with the Helsinki Declaration of 1975, as revised in 2000 [5]. Informed consent was obtained from all patients for being included in the study.

## Conflicts of Interest

The authors declare no conflicts of interest.

## Supporting information


Table S1.

Figure S1.
Figure S2.
Figure S3.
Figure S4.

## Data Availability

The data that support the findings of this study are available from the corresponding author, P.V., upon reasonable request.
