# Supplementary material for: Neonatal Cholestasis Progressing to a Multisystem Syndrome With Liver Cirrhosis in Two Siblings With FARSA Deficiency: An Evolving Hepatological Phenotype
Source: JIMD Rep. 2025 Apr 4;66(3):e70013. doi: 10.1002/jmd2.70013 (PMC11971029; doi:10.1002/jmd2.70013)
Supplement: Supplementary file 1 — Table S1. [file JMD2-66-e70013-s002.docx]

**Table S1. Detail of clinical features in FARS1 patients.**

Detail of clinical phenotype in FARS1 patients described here (P1 girl & P2 boy) and in literature were summarized in the table. Symbol X indicates the presence of a clinical feature in the patient. Grey squares reflect ‘negativity for symptoms’, white squares reflect ‘not mentioned, probably absent’. Symbol ° indicates that investigation was not assessed’. One patient in Zadjali et al. (P6) was excluded because of severe post-meningitis and brain herniation sequellae. (^1^ Seizures most probably due to hypoglycemia and hypocalcemia. ^2^ Stroke due to right internal carotid artery occlusion. ^3^ Subarachnoidal hemorrhage caused by a ruptured aneurysm, complicated by cerebral infarction because of post-SAH vasospasm. ^4^ Brain hemorrhage probably secondary to Lovenox therapy. ^5^ Dysmorphic features typically overlap with Marfan syndrome including marfanoid body habitus (i.e. short trunk), micrognathia, elfin-like face, arachnodactyly, joint hyperextensibility and dental crowding. ^6^ Other malformations were frontal bossing, deep and narrow-set eyes, prominent forehead, full cheeks, small nose, myopathic facies, hindfoot valgus, congenital hip dislocation.)

|  |  | | | FARSA (n=13) | | | | | | | | |  | |  | |  | |  | | |  | | |  | |  | | FARSB (n=18) | | | | | | | |  | |  | |  | |  | |  | | |  |
| --- | --- | --- | --- | --- | --- | --- | --- | --- | --- | --- | --- | --- | --- | --- | --- | --- | --- | --- | --- | --- | --- | --- | --- | --- | --- | --- | --- | --- | --- | --- | --- | --- | --- | --- | --- | --- | --- | --- | --- | --- | --- | --- | --- | --- | --- | --- | --- | --- |
|  | | P1 (girl) | P2 (boy) | | P1 Krenke et al. | P1 Schuch et al. | P2 Schuch et al. | P3 Schuch et al. | P1 Charbit-H. et al. | P2 Charbit-H. et al. | P3 Charbit-H. et al. | P4 Charbit-H. et al. | | P1 Kim et al. | | P1 Xu & Sun et al. | | P1 Guo et al. | | P1 Xu et al. | P2 Xu et al. | | P3 Xu et al. | P4 Xu et al. | | P5 Xu et al. | | P1 Antonellis et al. | | P1 Zadjali et al. | P2 Zadjali et al. | P3 Zadjali et al. | P4 Zadjali et al. | P5 Zadjali et al. | P6 Zadjali et al. | P7 Zadjali et al. | | P8 Zadjali et al. | | P4 Schuch et al. | | P5 Schuch et al. | | P1 Karimzadeh et al. | | P2 Karimzadeh et al. |  |  |
| Interstitial lung disease | | X | X | | X | X | X | X | X | X | X | X | | X | | X | | X | | X | X | | X | X | | X | | X | | X | X | X | X | X | X | X | | X | | X | | X | | X | |  |  |  |
| Cystic lung disease | |  |  | | X | X |  | X | X |  | X | X | |  | | X | |  | | X |  | | X |  | |  | | X | |  |  |  |  |  |  |  | |  | | X | | X | | X | |  |  |  |
| Cholesterol pneumonitis (lung biopsy) | | ° | ° | | X | X | X | X | X |  | X |  | |  | |  | |  | | X | X | | X | X | | X | | ° | | ° | ° | ° | ° | ° | ° | ° | | ° | | X | | X | | ° | | ° |  |  |
| Pulmonary alveolar proteinosis | |  |  | |  |  | X |  |  | X |  |  | |  | |  | |  | |  |  | | X |  | |  | |  | |  |  |  |  |  |  |  | |  | |  | |  | |  | |  |  |  |
| Intra-alveolar hemorrhage | |  |  | |  | X |  |  | X |  |  |  | |  | |  | |  | |  |  | |  |  | |  | |  | |  |  |  |  |  |  |  | |  | |  | |  | |  | |  |  |  |
| Chronic cough | |  |  | | X |  |  | X | X | X |  |  | |  | |  | |  | |  |  | |  |  | |  | | X | |  |  |  | X |  |  |  | | X | |  | | X | |  | |  |  |  |
| Recurrent spontaneous pneumothorax | |  |  | |  |  |  |  |  |  |  |  | |  | |  | |  | | X | X | |  |  | |  | |  | |  |  |  |  |  |  |  | |  | |  | |  | | X | |  |  |  |
| Digital clubbing | | X |  | | X | X |  | X | X | X |  |  | |  | |  | |  | |  |  | | X | X | |  | |  | |  |  |  |  |  |  |  | |  | | X | | X | |  | |  |  |  |
| (Neonatal) hypotonia | | X | X | | X | X | X |  |  |  |  |  | | X | | X | | X | | X | X | | X | X | | X | | X | |  |  |  |  |  |  |  | |  | |  | |  | |  | |  |  |  |
| Decreased muscle mass | | X |  | | X |  |  | X |  |  |  |  | |  | |  | | X | | X |  | | X |  | |  | |  | | X | X | X | X | X | X | X | | X | |  | |  | |  | |  |  |  |
| Abnormal muscle histology | | ° | ° | | ° | X | ° | ° | ° | ° | ° | ° | | ° | | ° | | ° | | X | X | |  | ° | |  | | X | | ° | ° | ° | ° | ° | ° | ° | | ° | | ° | | ° | |  | |  |  |  |
| Delayed motor development | | X |  | | X | X | X |  |  |  | X | X | | X | |  | | X | | X | X | | X |  | | X | | X | | X | X | X | X | X | X | X | |  | |  | |  | |  | |  |  |  |
| Speech delay | |  |  | |  | X | X |  |  |  |  |  | | X | |  | | X | |  |  | | X |  | |  | |  | | X | X | X | X |  | X |  | |  | |  | |  | |  | |  |  |  |
| Intellectual disability, learning difficulties | |  |  | |  |  | X |  |  |  | X | X | | X | |  | |  | |  |  | | X |  | |  | |  | | X | X | X | X | X | X | X | | X | |  | |  | |  | |  |  |  |
| Headache, migraine | |  |  | |  | X |  |  |  |  |  |  | |  | |  | |  | |  |  | |  |  | |  | |  | | X | X | X | X | X |  | X | |  | | X | |  | | X | |  |  |  |
| Seizures | |  |  | |  |  |  |  |  |  |  |  | |  | |  | |  | |  | X | |  |  | |  | | X^1^ | |  |  |  | X |  |  |  | |  | |  | |  | | X | |  |  |  |
| Microcephaly | |  |  | |  |  |  |  | X | X | X | X | |  | |  | |  | |  | X | |  |  | | X | |  | | X | X | X | X | X | X | X | | X | |  | |  | |  | |  |  |  |
| Extrapyramidal symptoms | |  |  | |  |  |  |  |  |  |  |  | |  | |  | |  | |  |  | |  |  | |  | |  | | X |  |  |  |  |  |  | |  | |  | |  | | X | | X |  |  |
| Brain cysts (MRI) | |  | X | | X |  |  | X |  |  |  |  | |  | |  | |  | |  |  | |  | ° | |  | |  | |  |  |  |  |  |  |  | |  | |  | | ° | |  | |  |  |  |
| Brain calcifications (MRI) | |  |  | | X |  |  |  |  |  |  |  | | X | | X | |  | | X | X | | X | ° | |  | |  | | X | X | X | X | X | X | X | | X | |  | | ° | | X | | X |  |  |
| White matter and gliotic lesions (MRI) | |  | X | |  | X |  |  | X |  | X | X | |  | |  | |  | |  | X | | X | ° | |  | |  | |  |  |  |  |  |  |  | |  | | X | | ° | |  | |  |  |  |
| Brain atrophy (MRI) | |  |  | |  |  |  |  |  |  | X |  | | X | |  | |  | |  |  | |  | ° | |  | | X | |  |  |  |  |  |  |  | |  | |  | | ° | |  | |  |  |  |
| Brain aneurysm or elongated arteries (MRI) | |  |  | |  | X |  |  |  |  |  | X | |  | |  | |  | | X | X | |  | ° | |  | |  | |  |  |  |  |  |  |  | |  | |  | | ° | |  | |  |  |  |
| Brain hemorrhage / stroke | |  |  | |  |  |  |  |  |  | X^2^ | X^3^ | |  | |  | |  | |  | X | |  |  | |  | | X^4^ | |  |  |  |  |  |  |  | |  | |  | |  | |  | |  |  |  |
| Hydrocephalus / ventriculomegaly | |  |  | |  |  |  |  |  |  |  |  | |  | | X | |  | |  |  | | X |  | |  | | X | |  |  |  | X |  |  |  | |  | |  | |  | |  | |  |  |  |
| Hypopituitarism | |  |  | | X |  |  |  |  |  |  |  | |  | |  | |  | |  |  | |  |  | |  | |  | |  |  |  |  |  |  |  | |  | |  | |  | |  | |  |  |  |
| Hepatomegaly, splenomegaly | | X | X | | X | X | X |  | X |  | X | X | | X | |  | | X | |  | X | |  | X | | X | |  | |  |  |  |  |  |  |  | |  | |  | |  | |  | |  |  |  |
| Elevated transaminases (AST, ALT) | |  | X | | X | X | X | X |  |  |  |  | | X | | X | | X | |  |  | | X | X | |  | | X | |  |  |  |  |  |  |  | |  | |  | |  | |  | |  |  |  |
| Elevated cholestasis parameters (gGT, bili) | |  | X | | X |  |  | X |  |  |  |  | |  | | X | | X | |  |  | |  |  | |  | | X | |  |  |  |  |  |  |  | |  | |  | |  | |  | |  |  |  |
| Liver steatosis, fibrosis, cirrhosis | | X | X | | X | X | X | ° | X | X | X | X | | X | |  | |  | | X | X | |  |  | | X | | X | | ° | ° | ° | ° | ° | ° | ° | | ° | |  | |  | |  | |  |  |  |
| (Neonatal) jaundice | | X | X | |  |  |  |  |  |  |  |  | |  | |  | | X | |  |  | |  |  | |  | |  | |  |  |  |  |  |  |  | |  | |  | |  | |  | |  |  |  |
| Feeding intolerance / difficulties | |  | X | | X |  | X |  |  |  |  |  | | X | |  | |  | |  |  | |  |  | |  | | X | |  |  |  | X |  |  |  | |  | |  | |  | |  | |  |  |  |
| Recurrent vomiting and/or diarrhea | |  | X | | X |  | X |  | X | X |  |  | | X | |  | |  | |  |  | | X | X | | X | |  | |  |  |  |  |  |  |  | |  | |  | | X | |  | |  |  |  |
| Inguinal hernia | |  | X | | X |  |  |  |  |  |  |  | |  | |  | |  | | X |  | |  |  | |  | | X | |  |  |  |  |  |  |  | |  | |  | |  | |  | |  |  |  |
| Intestinal malrotation | |  |  | |  |  |  |  |  |  |  |  | |  | |  | |  | |  | X | | X |  | |  | |  | |  |  |  |  |  |  |  | |  | |  | |  | |  | |  |  |  |
| Gastroesophageal reflux | |  |  | |  |  |  |  |  |  |  |  | |  | |  | |  | |  |  | |  |  | | X | | X | |  |  |  |  |  |  |  | |  | |  | |  | |  | |  |  |  |
| Gastroesophageal varices / hemorrhage | | X | X | |  |  |  |  |  |  |  |  | |  | |  | |  | |  |  | |  |  | |  | | X | |  |  |  |  |  |  |  | |  | |  | |  | |  | |  |  |  |
| Failure to thrive, poor weight gain | | X | X | | X | X | X | X | X | X | X | X | | X | | X | | X | | X | X | |  | X | | X | | X | | X | X | X | X | X | X | X | | X | | X | | X | | X | |  |  |  |
| Growth hormone resistance/deficiency | |  |  | |  | X |  | X |  |  |  |  | |  | |  | |  | |  |  | |  |  | |  | |  | |  |  |  |  |  |  |  | |  | |  | |  | |  | |  |  |  |
| Short stature | | X | X | | X | X |  | X | X | X | X | X | |  | | X | |  | |  |  | |  |  | |  | |  | | X | X | X | X | X | X | X | | X | |  | |  | |  | |  |  |  |
| Structural heart or vessel defects | |  | X | |  | X |  | X |  |  |  |  | |  | |  | |  | |  |  | |  |  | |  | |  | |  |  |  |  |  |  |  | |  | |  | | X | |  | |  |  |  |
| Arterial hypertension | |  |  | |  |  |  |  |  |  |  |  | |  | |  | |  | | X | X | |  |  | |  | |  | |  |  |  |  |  |  |  | |  | |  | |  | |  | |  |  |  |
| Vesicoureteral reflux | |  |  | | X |  |  |  |  |  |  |  | |  | |  | |  | |  |  | |  | X | |  | |  | |  |  |  |  |  |  |  | |  | |  | |  | |  | |  |  |  |
| Proteinuria | |  | X | |  |  |  |  |  |  |  |  | | X | |  | |  | |  |  | | X |  | | X | |  | |  |  |  |  |  |  |  | |  | |  | | X | |  | |  |  |  |
| Renal artery stenosis | |  |  | |  |  |  |  |  |  |  |  | |  | |  | |  | | X |  | |  |  | |  | |  | |  |  |  |  |  |  |  | |  | |  | |  | |  | |  |  |  |
| Hyperphosphaturia | |  |  | |  |  | X |  |  |  |  |  | |  | |  | |  | |  |  | |  |  | |  | |  | |  |  |  |  |  |  |  | |  | |  | |  | |  | |  |  |  |
| Glomerulosclerosis (renal biopsy) | | ° | ° | | ° | ° | ° | ° | ° | ° | ° | ° | | ° | |  | |  | | ° | ° | | ° | ° | | X | | ° | | ° | ° | ° | ° | ° | ° | ° | | ° | | ° | | ° | |  | |  |  |  |
| Tubulopathy | |  | X | |  |  | X |  |  |  |  |  | | X | |  | |  | |  |  | |  |  | |  | |  | |  |  |  |  |  |  |  | |  | |  | |  | |  | |  |  |  |
| Nephrolithiasis | |  |  | |  |  |  | X |  |  |  |  | |  | | X | |  | |  |  | |  |  | |  | |  | |  |  |  |  |  |  |  | |  | |  | |  | |  | |  |  |  |
| Renal hyperechogenicity (ultrasound) | |  |  | |  |  |  |  |  |  |  |  | | X | |  | |  | |  |  | |  |  | |  | | X | |  |  |  |  |  |  |  | |  | |  | |  | |  | |  |  |  |
| Abnormal eye movement, nystagmus | |  |  | |  |  | X |  |  |  |  |  | |  | |  | |  | |  |  | |  |  | |  | | X | |  |  |  |  |  |  |  | |  | |  | |  | |  | |  |  |  |
| Sensorineural hearing impairment | |  |  | |  |  |  | X |  |  |  |  | |  | |  | |  | |  |  | |  |  | |  | |  | |  |  |  |  |  |  |  | |  | | X | |  | |  | |  |  |  |
| (Congenital) hypothyroidism | |  |  | | X |  |  |  |  |  |  |  | | X | |  | | X | | X |  | |  |  | |  | |  | |  |  |  |  |  |  |  | |  | |  | |  | |  | |  |  |  |
| Anemia (microcytic) | |  | X | | X |  |  |  |  |  |  |  | | X | | X | |  | |  |  | |  |  | |  | | X | | X | X | X | X | X | X | X | | X | |  | |  | |  | |  |  |  |
| Thrombocytopenia | | X | X | |  |  |  |  |  |  |  |  | | X | |  | |  | |  |  | |  |  | |  | | X | |  |  |  |  |  |  |  | |  | |  | |  | |  | |  |  |  |
| Leukopenia, neutropenia | | X |  | |  |  |  |  |  |  |  |  | | X | |  | |  | |  |  | |  |  | |  | | X | |  |  |  |  |  |  |  | |  | |  | |  | |  | |  |  |  |
| History of DVT | |  | X | |  |  |  |  |  |  |  |  | |  | |  | |  | |  |  | |  |  | |  | | X | |  |  |  |  |  |  |  | |  | |  | |  | |  | |  |  |  |
| Hypoalbuminemia | | X | X | | X | X | X | X | X | X | X | X | |  | | X | | X | |  |  | |  |  | | X | | X | | X | X |  |  |  |  |  | |  | |  | | X | |  | |  |  |  |
| Hypocalcemia | |  |  | |  |  |  |  |  |  |  |  | |  | | X | |  | |  |  | |  |  | |  | | X | |  |  |  |  |  |  |  | |  | |  | |  | |  | |  |  |  |
| Hypertriglyceridemia | |  |  | | X |  |  |  |  |  |  |  | |  | |  | |  | |  |  | |  |  | | X | |  | |  |  |  |  |  |  |  | |  | |  | |  | |  | |  |  |  |
| Hypoglycemia | |  | X | |  |  |  |  |  |  |  |  | |  | |  | |  | |  |  | |  |  | |  | | X | |  |  |  |  |  |  |  | |  | |  | |  | |  | |  |  |  |
| Neonatal rachitis | | X |  | |  |  |  |  |  |  |  |  | |  | |  | |  | |  |  | |  |  | |  | | X | |  |  |  |  |  |  |  | |  | |  | |  | |  | |  |  |  |
| Marfan-like dysmorphism^5^ | |  |  | | X | X |  | X | X | X | X | X | |  | |  | |  | | X | X | | X |  | |  | | X | |  |  |  |  |  |  |  | |  | |  | |  | |  | |  |  |  |
| Other malformations^6^ | |  |  | |  |  |  |  |  |  |  |  | |  | | X | | X | |  |  | |  | X | | X | | X | |  |  |  |  |  |  |  | |  | |  | |  | |  | |  |  |  |
| Chest deformity (pectus excavatum/carinatum) | |  |  | | X | X |  | X |  |  | X | X | |  | |  | |  | | X |  | | X |  | |  | |  | |  |  |  |  |  |  |  | |  | |  | | X | |  | |  |  |  |
| Scoliosis | |  |  | | X |  |  |  |  |  |  |  | |  | |  | |  | | X | X | |  |  | |  | |  | |  |  |  |  |  |  |  | |  | |  | |  | |  | |  |  |  |
| Osteopenia | |  | X | |  | X | X |  |  |  |  |  | |  | |  | |  | | X |  | | X |  | |  | | X | | X | X | X | X |  |  |  | |  | |  | |  | |  | |  |  |  |
| Poor wound healing | |  | X | |  | X |  |  |  |  |  |  | |  | |  | |  | | X |  | |  |  | |  | |  | |  |  |  |  |  |  |  | |  | |  | |  | |  | |  |  |  |
| Abnormal subcutaneous fat tissue distribution | |  |  | | X |  | X |  |  |  |  |  | |  | |  | |  | |  |  | | X | X | | X | |  | |  |  |  |  |  |  |  | |  | |  | |  | |  | |  |  |  |
