# Supplementary material for: Neonatal Cholestasis Progressing to a Multisystem Syndrome With Liver Cirrhosis in Two Siblings With FARSA Deficiency: An Evolving Hepatological Phenotype
Source: JIMD Rep. 2025 Apr 4;66(3):e70013. doi: 10.1002/jmd2.70013 (PMC11971029; doi:10.1002/jmd2.70013)
Supplement: Supplementary file 2 — Figure S1. Figure S2. Figure S3. Figure S4. [file JMD2-66-e70013-s001.pdf]

SUPPLEMENT. Additional Tables and Figures.

Figure S1. Family tree.

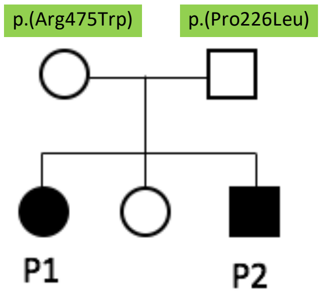

Figure S2. Growth curves of patient 1 (2.1) and patient 2 (2.2)

2.1. Growth curve P1.

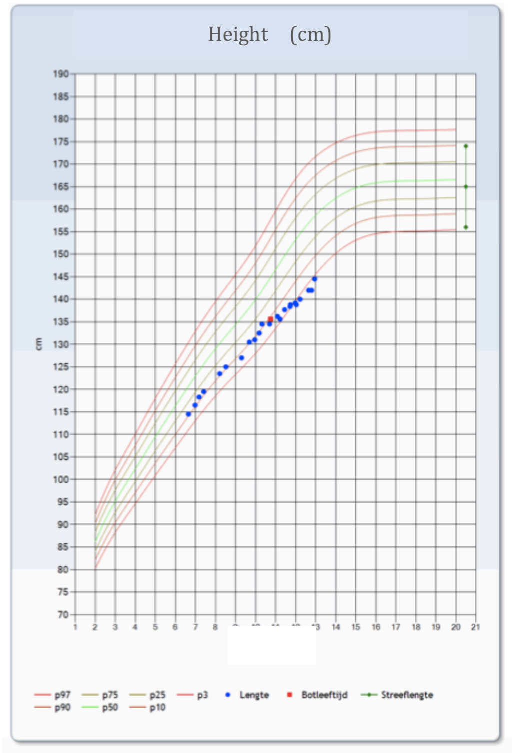

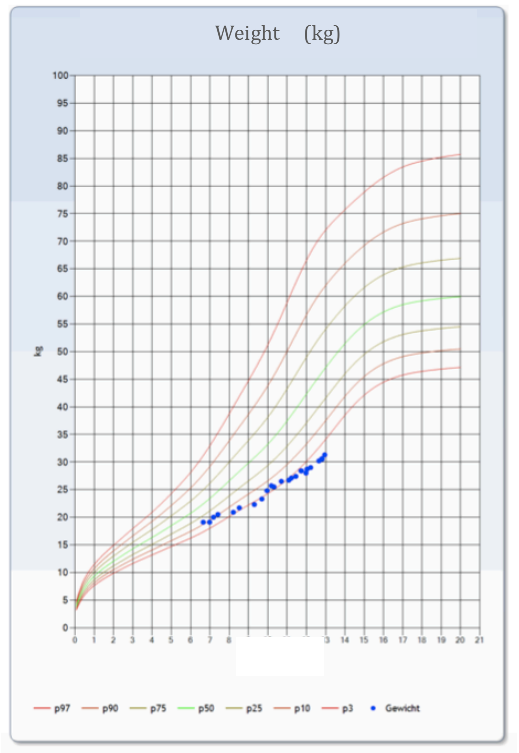

2.2. Growth curve P2.

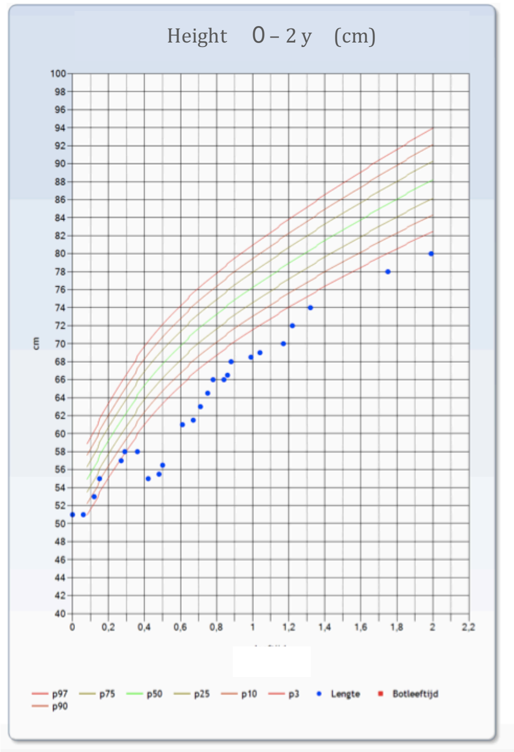

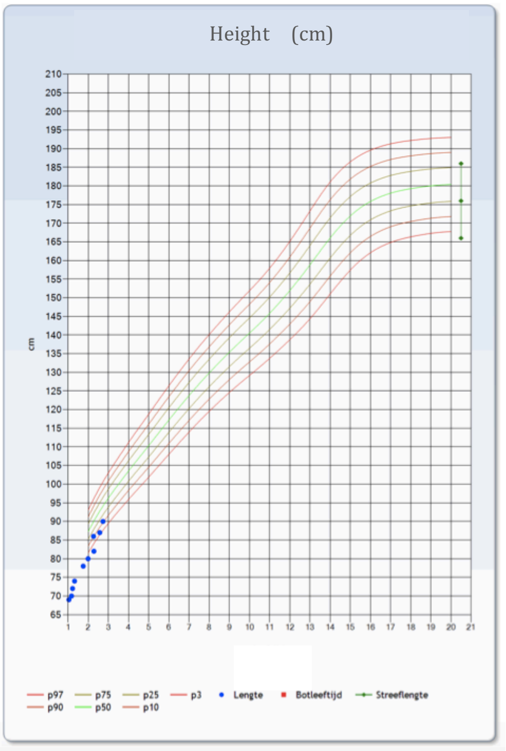

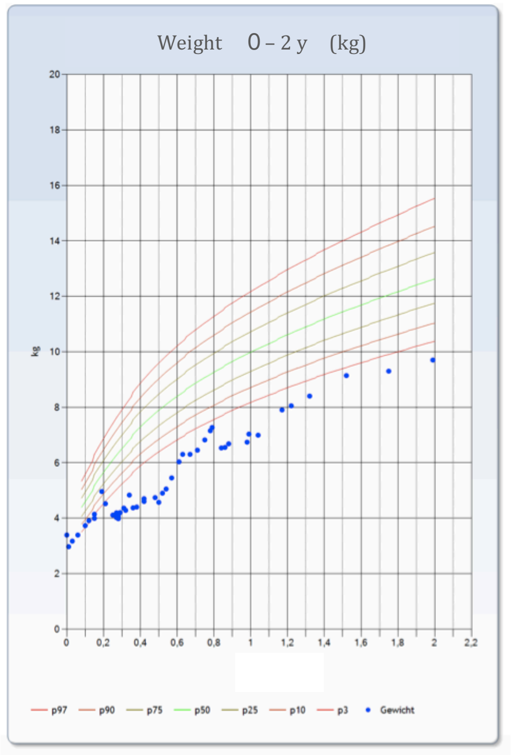

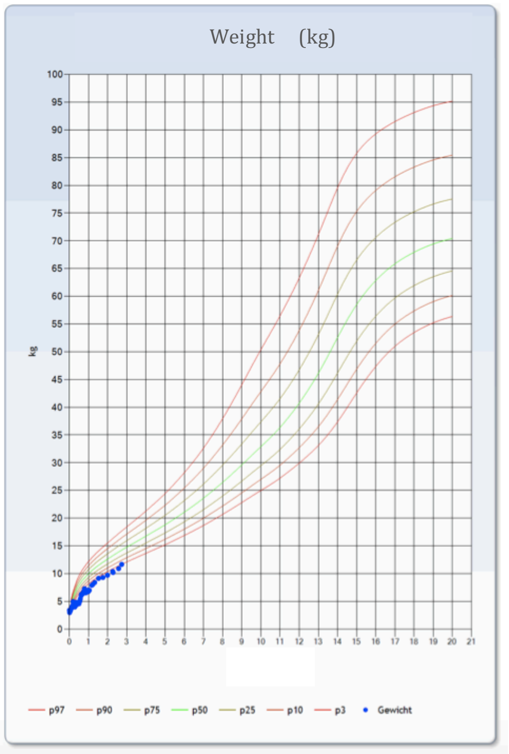

Figure S3. Overview of lab results in patient 1.

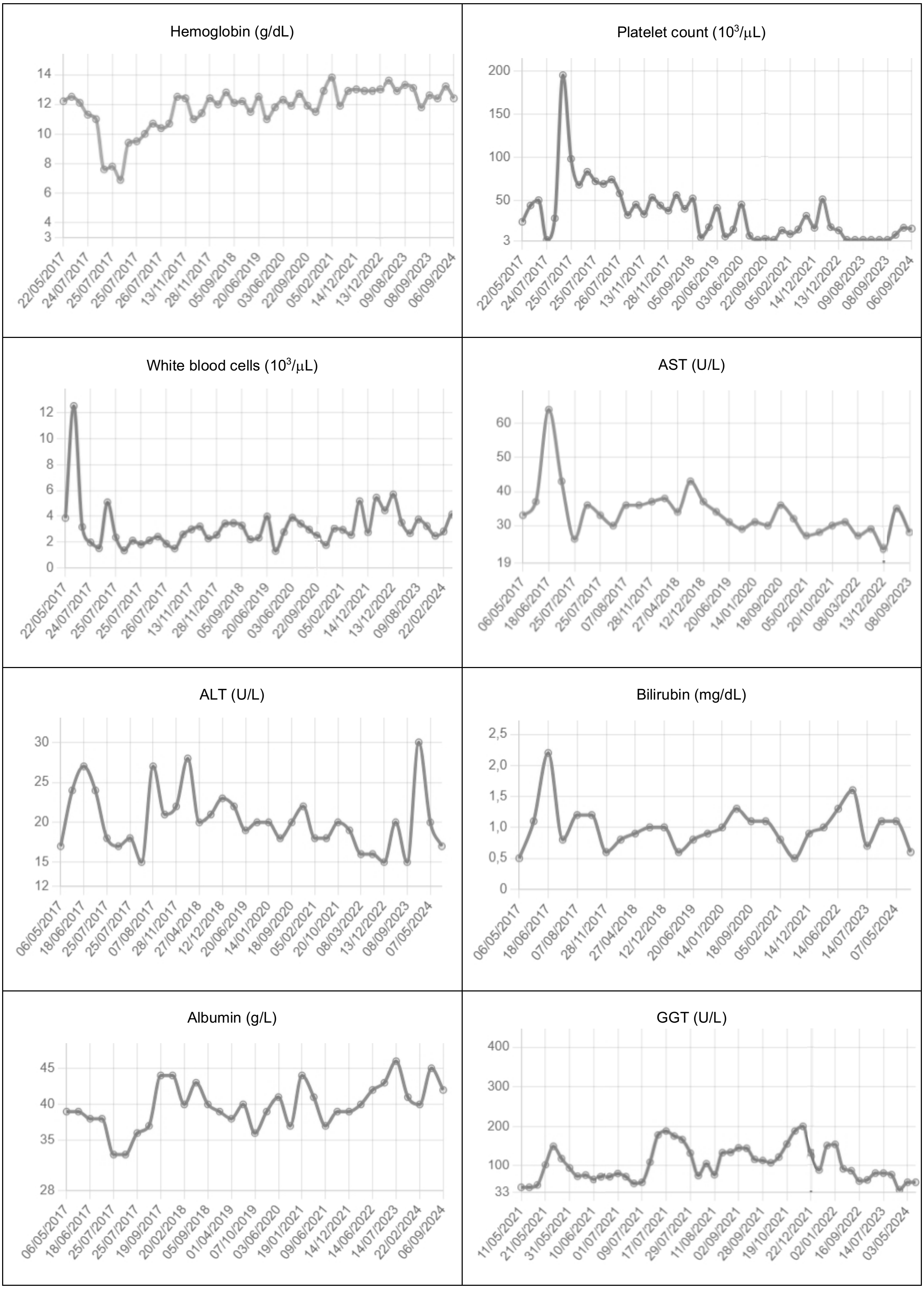

Figure S4. Overview of lab results in patient 2 during initial hospitalisations and further follow-up.

4.1. Laboratory findings during initial hospitalization. Transfusions with red blood cells and platelets were shown on the appropriate chart (A-B). Severe infectious episodes were due to 1) a localized *Stenotrophonomonas* catheter site infection, 2) a *Candida parapsilosis* catheter-related blood-stream infection and COVID-19, 3) CMV enterocolitis and 4) *E. coli* urosepsis (D). Several albumin infusions were needed because of recurrent hypoalbuminemia (H).

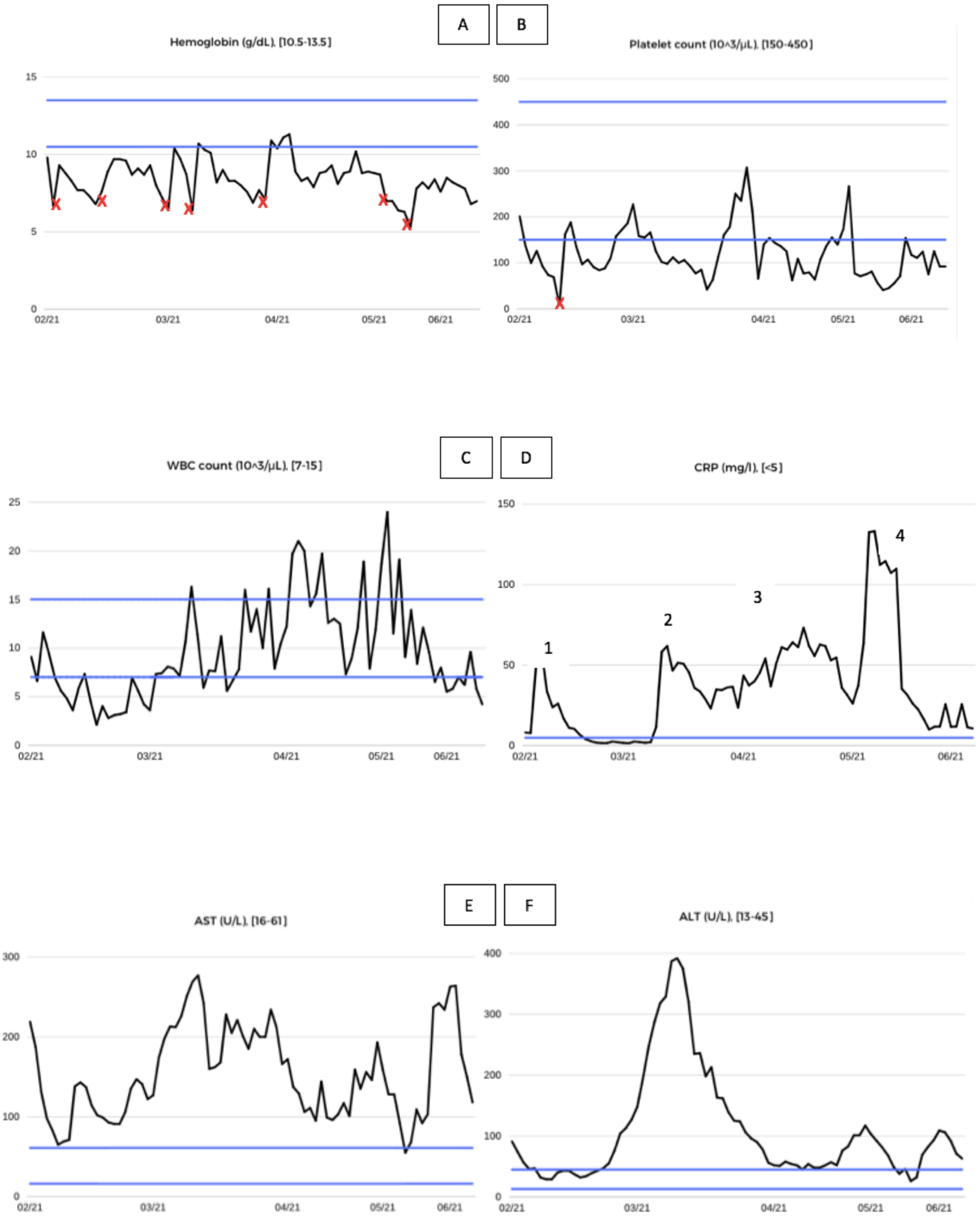

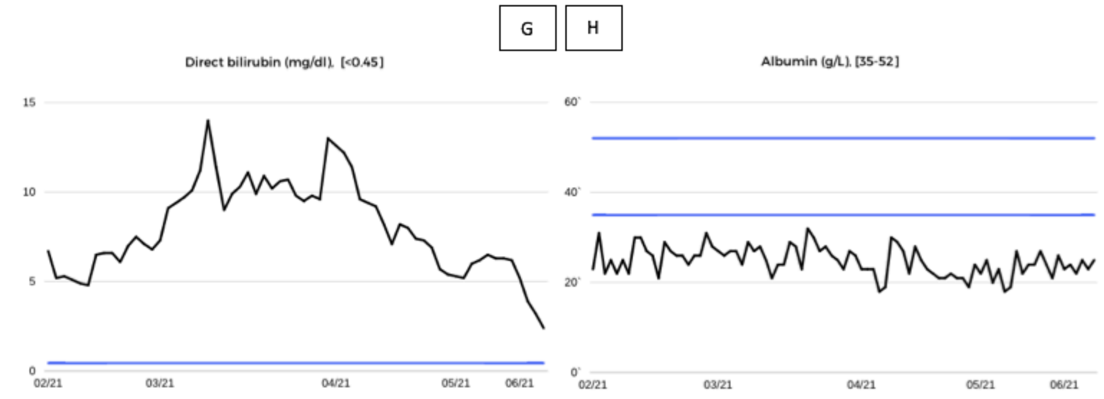

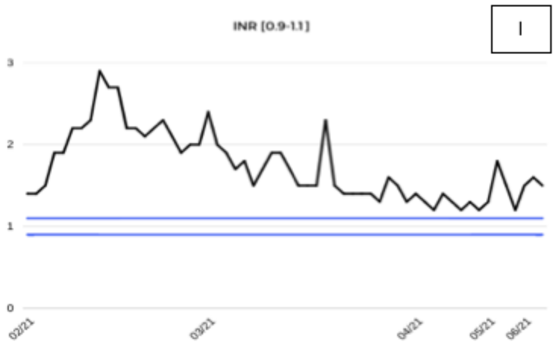

4.2. Overview of lab results during further follow-up.

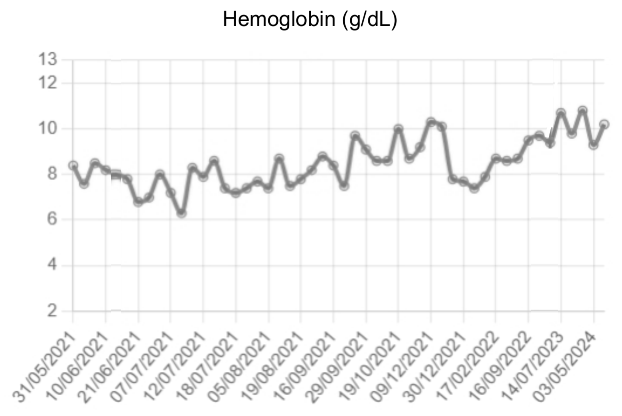

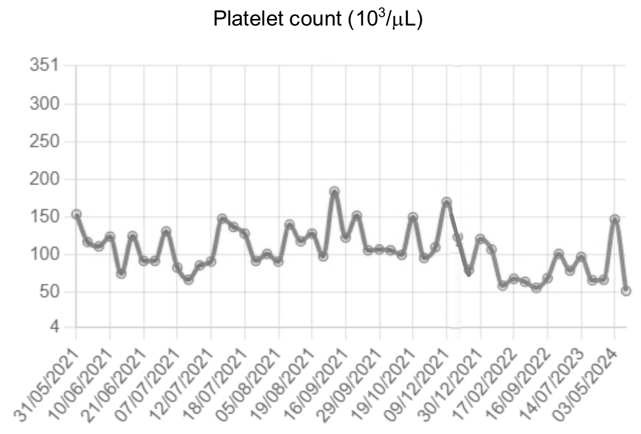

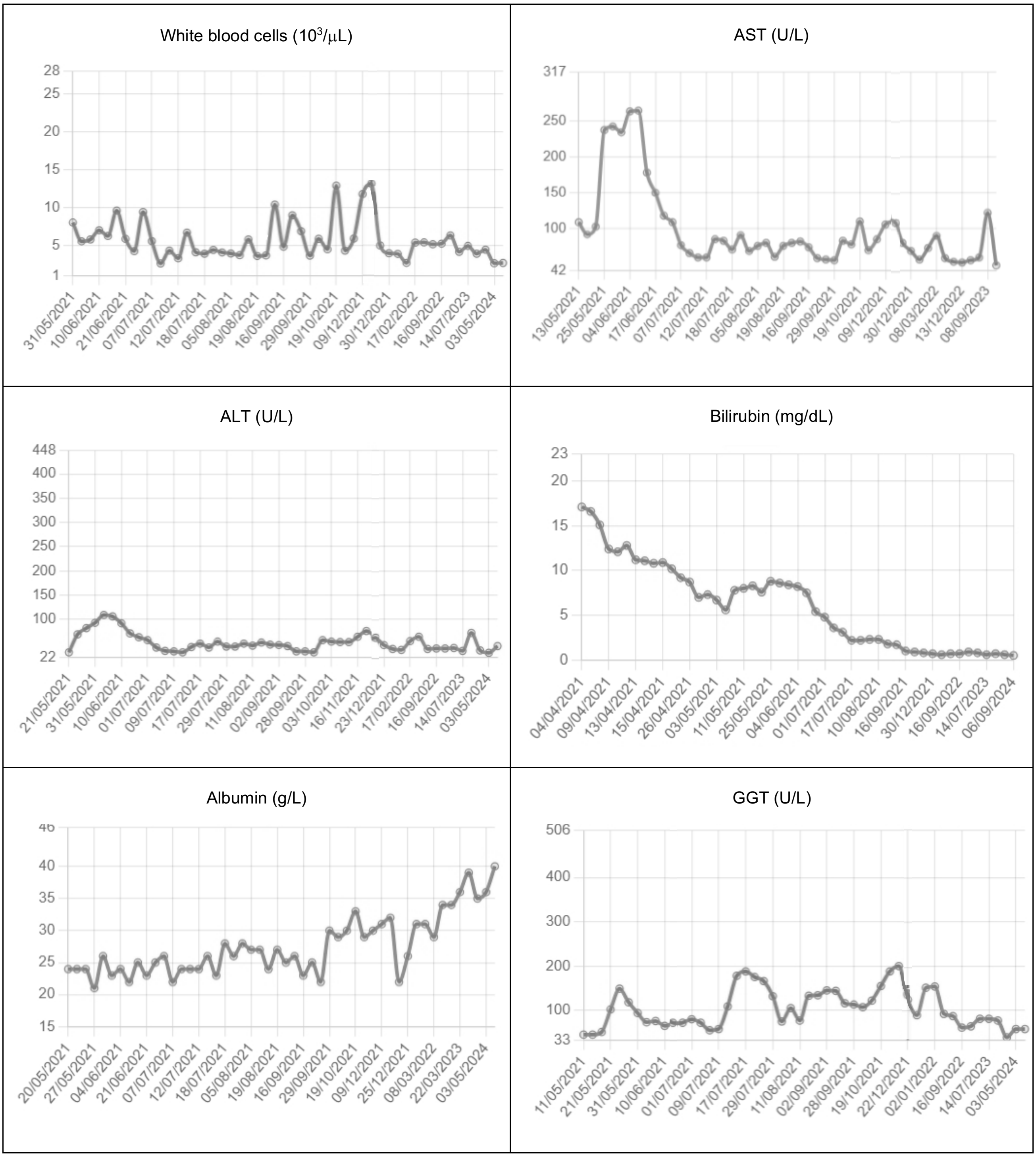
White blood cells ($10^3/\mu L$)
28 25 20 15 10 5 1
31/05/2021 10/06/2021 21/06/2021 07/07/2021 12/07/2021 18/07/2021 05/08/2021 19/08/2021 16/09/2021 29/09/2021 19/10/2021 09/12/2021 30/12/2021 17/02/2022 16/09/2022 14/07/2023 03/05/2024
AST (U/L)
317 250 200 150 100 42
13/05/2021 25/05/2021 04/06/2021 17/06/2021 07/07/2021 12/07/2021 18/07/2021 05/08/2021 19/08/2021 16/09/2021 29/09/2021 19/10/2021 09/12/2021 30/12/2021 08/03/2022 13/12/2022 08/09/2023
ALT (U/L)
448 400 350 300 250 200 150 100 22
21/05/2021 31/05/2021 10/06/2021 01/07/2021 09/07/2021 17/07/2021 29/07/2021 11/08/2021 02/09/2021 28/09/2021 03/10/2021 16/11/2021 23/12/2021 17/02/2022 16/09/2022 14/07/2023 03/05/2024
Bilirubin (mg/dL)
23 20 15 10 5 0
04/04/2021 09/04/2021 13/04/2021 15/04/2021 26/04/2021 03/05/2021 11/05/2021 25/05/2021 04/06/2021 01/07/2021 17/07/2021 10/08/2021 16/09/2021 30/12/2021 16/09/2022 14/07/2023 06/09/2024
Albumin (g/L)
40 35 30 25 20 15
20/05/2021 27/05/2021 04/06/2021 21/06/2021 07/07/2021 12/07/2021 18/07/2021 05/08/2021 19/08/2021 16/09/2021 29/09/2021 19/10/2021 09/12/2021 25/12/2021 08/03/2022 22/03/2023 03/05/2024
GGT (U/L)
506 400 300 200 100 33
11/05/2021 21/05/2021 31/05/2021 10/06/2021 01/07/2021 09/07/2021 17/07/2021 29/07/2021 11/08/2021 02/09/2021 28/09/2021 19/10/2021 22/12/2021 02/01/2022 16/09/2022 14/07/2023 03/05/2024
